# Meta-Analysis of the Effect of Bowel Preparation on Adenoma Detection: Early Adenomas Affected Stronger than Advanced Adenomas

**DOI:** 10.1371/journal.pone.0154149

**Published:** 2016-06-03

**Authors:** Michael C. Sulz, Arne Kröger, Meher Prakash, Christine N. Manser, Henriette Heinrich, Benjamin Misselwitz

**Affiliations:** 1 Division of Gastroenterology and Hepatology, University Hospital Zurich, Zurich, Switzerland; 2 Division of Gastroenterology and Hepatology, Kantonsspital St. Gallen, St. Gallen, Switzerland; 3 See-Spital Horgen, 8801, Horgen, Switzerland; Virginia Tech University, UNITED STATES

## Abstract

**Background and Aims:**

Low-quality bowel preparation reduces efficacy of colonoscopy. We aimed to summarize effects of bowel preparation on detection of adenomas, advanced adenomas and colorectal cancer.

**Methods:**

A systematic literature search was performed regarding detection of colonic lesions after normal and low-quality bowel preparation. Reported bowel preparation quality was transformed to the Aronchick scale with its qualities “excellent”, “good”, “fair”, “poor”, and “insufficient” or “optimal” (good/excellent), “suboptimal” (fair/poor/insufficient), “adequate” (good/excellent/fair) and “inadequate” (poor/insufficient). We identified two types of studies: i) Comparative studies, directly comparing lesion detection according to bowel preparation quality, and ii) repeat colonoscopy studies, reporting results of a second colonoscopy after previous low-quality preparation.

**Results:**

The detection of early adenomas was reduced with inadequate vs. adequate bowel preparation (Odds Ratio (OR) 0.53, CI: 0.46–0.62, p<0.001). The advanced adenomas were affected less in comparison (0.74, CI: 0.62–0.87, p<0.001). The large number of subjects considered in the present meta-analysis resulted in smaller confidence intervals compared to earlier studies. Classifying the bowel-preparation quality as suboptimal vs. optimal led to the same qualitative conclusion (OR: 0.81, CI: 0.74–0.89, p<0.001 for early adenomas, OR: 0.94, CI: 0.87–1.01, n.s. for advanced adenomas). Bowel preparation was equally important for right-sided/ flat/ serrated vs. other lesions in most observational studies but more relevant in some repeat colonoscopy studies; data regarding carcinoma detection were insufficient.

**Conclusion:**

Inadequate bowel preparation affects detection of early colonic lesions stronger than advanced lesions.

## Introduction

Colorectal cancer (CRC) remains the second most common cancer in women and the third most common in men [1]. In industrialized countries the lifetime incidence for patients at average risk is approximately 5%, and more than 600’000 patients die from this cancer every year [2].

CRC incidence and mortality can be reduced by endoscopic screening since precancerous lesions (early and advanced adenomas) can be detected and removed during the intervention [3,4]. In a large randomized study one-time screening with sigmoidoscopy resulted in a 23% decrease in CRC incidence and a 31% decrease in CRC mortality after a follow up of 11 years [3]. The protective effect of colonoscopy so far has not been tested in randomized trials but should exceed the effect of sigmoidoscopy since the whole colon is visualized. Nevertheless, colonoscopy is regarded as the most effective CRC screening strategy by gastroenterologists and professional organizations [2,5,6].

A high quality of colonoscopy is decisive for maximum protection from CRC. Interval carcinoma refer to carcinoma detected before the recommended surveillance interval and might be responsible for up to 10% of all CRCs [7–9]. Adenoma detection rate (ADR) is inversely correlated with interval cancer development [9,10] and widely used as a surrogate for the quality of colonoscopy [11]. Many factors including experience of the endoscopist, withdrawal time, and quality of bowel preparation are associated with ADR [11].

Suboptimal bowel preparation has been reported in as much as 20% of all colonoscopies [12,13], possibly reducing ADR. The best strategy after such a colonoscopy remains unclear: Even though poor bowel preparation reduces protection from CRC, an immediate repetition of colonoscopy clearly offers less benefit then the original intervention. Clarity regarding effects of bowel preparation on differential detection of adenomas, advanced adenomas and CRC is needed to enable an informed decision regarding repetition of colonoscopy. Missing early colonic lesions will be inconsequential in the majority of cases since only a minority will ever transform to cancer. However, detection of advanced lesions will critically impact the future clinical course and detection of these lesions accounts for the largest impact of colonoscopy on CRC prevention. However, a previous meta-analysis demonstrated widely overlapping confidence intervals for the detection of early vs. advanced lesions [16].

We decided to perform another systematic review and meta-analysis regarding the effect of bowel preparation, considerably expanding the previous meta-analysis [16]. Our analysis revealed a stronger effect of bowel preparation on the detection of advanced vs. early colonic lesions.

## Materials and Methods

Between November 1^st^ and November 7^th^ 2014 we performed a systematic PubMed literature research regarding the impact of quality of bowel preparation on detection of lesions. The following search strategy was used: (Adenoma detection OR polyp detection) AND bowel preparation, colonoscopy AND Boston bowel preparation scale (BBPS), colonoscopy AND Ottawa scale, colonoscopy AND Aronchick scale, and colonoscopy AND tandem colonoscopy (S1 file). The abstracts of all publications were screened and potentially relevant papers retrieved. In addition, a search within the reference list of several publications including a recent meta-analysis [16] identified 3 additional relevant articles.

### Inclusion criteria

**Fig 1:**
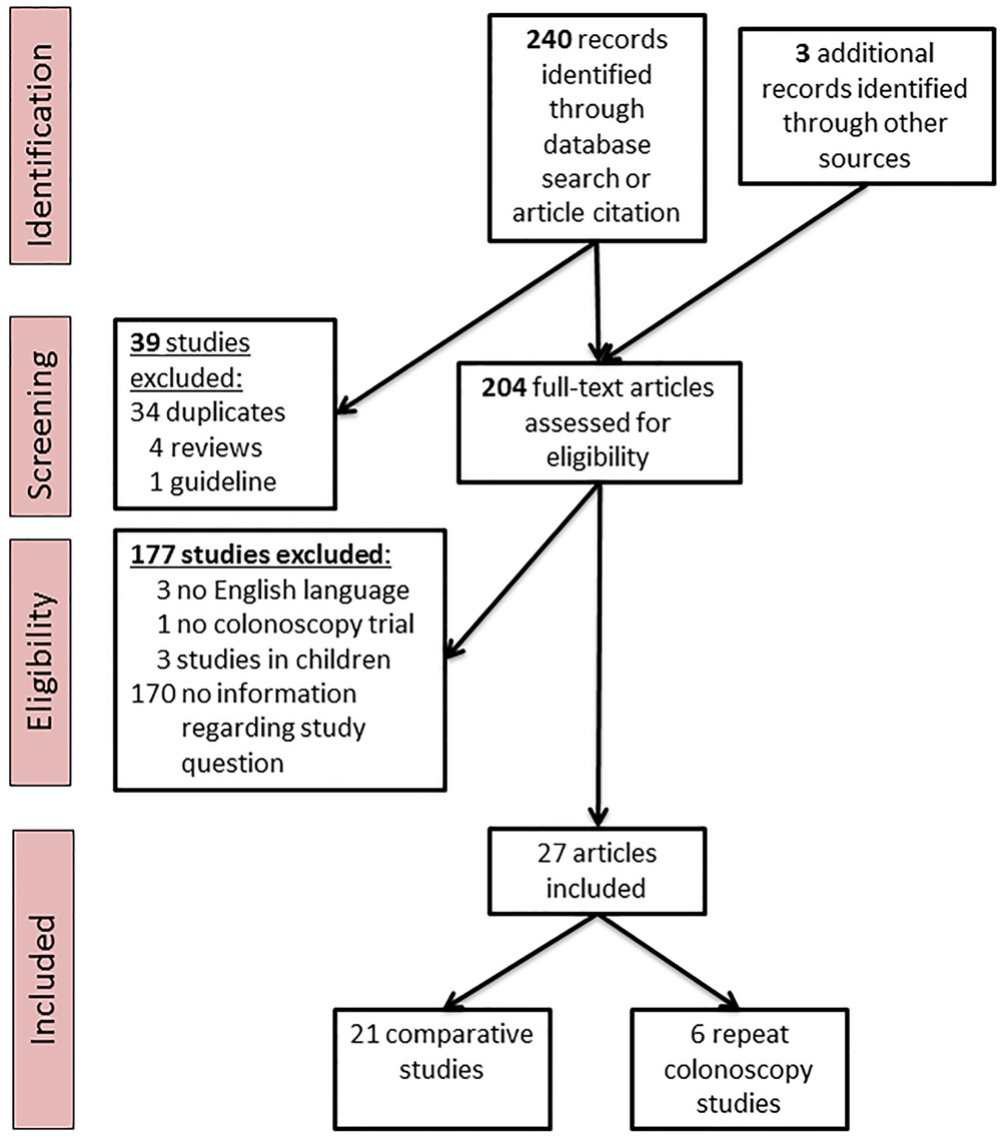
PRISMA flow diagram.

Our analysis identified two study types: Comparative studies (for which adenoma/polyp detection rates were compared according to bowel preparation quality within a given study population) and repeat-colonoscopy studies (for which after low-quality colonoscopy the investigation was repeated). The study selection process is shown in Fig 1. Independent sets inclusion criteria were defined: Comparative studies were included if the following criteria were met: i) bowel preparation was defined and reported. ii) adenoma or polyp detection was reported as raw numbers and/or odds ratios for at least two qualities of bowel preparation [16]. Repeat colonoscopy studies were included if: i) Colonoscopy was repeated for at least a fraction of patients, ii) bowel preparation was defined and reported for the first and second colonoscopy, iii) the first and the second colonoscopy reported lesion detection rates and/or miss rates (defined by the number of detected lesions in the second colonoscopy divided by the sum of lesion in both colonoscopies).

For a separate analysis addressing differential detection of flat/serrated and/or right-sided lesions studies were included if: i) Bowel preparation was defined and reported and ii) data for a comparison with either all polyps/adenomas and/or left-sided, pedunculated or non-serrated lesions was available.

Only studies for which an adult study population was evaluated by *complete* colonoscopy and only articles in English published before November 2014 were considered. If the same study population had been analyzed more than once only the latest analysis was included. Review and data extraction of each study was performed by two authors (BM and MCS or AK); discrepancies were resolved after discussions. All relevant data were retrieved from the original publications; the authors of the respective studies were not contacted in case of missing, incomplete or incomprehensible data.

Studies differed regarding their outcomes: Colonic lesions were either summarized as polyps, referring to protruding lesions detected during endoscopy without histological information or adenomas, for which histological confirmation was required. Similarly, advanced lesions were either summarized as advanced polyps, referring to lesions ≥1cm diameter without histological information or advanced adenomas. The latter category included lesions with villous/ tubulovillous or serrated histology irrespective of its size or tubular adenomas with a diameter ≥1cm. If data regarding both, adenoma and polyp detection, were available, only adenoma data were considered. We included data regarding polyp/adenoma detection using the following hierarchy: Odds Ratio (OR) derived from a multivariate analysis, OR from a univariate analysis, raw numbers.

### Analysis of bowel preparation

The Aronchick scale [17] was the most frequently used bowel preparation scale (Table 1) and the 5 preparation qualities “excellent”, “good”, “fair”, “poor”, and “insufficient” were used throughout this study. In addition we compared “optimal” (defined as “excellent” or “good”) with “suboptimal” (“fair”, “poor”, or “insufficient”) and “adequate” (“excellent”, “good”, or “fair”) with “inadequate” (“poor” or “insufficient”) preparations. BBPS was converted to the Aronchick scale using data from Lai et al. [18]. Only one publication used the Ottawa scale and due to limited data only conversion to the broad categories optimal/ suboptimal was possible [19]. No study used the Chicago scale. Unique scales were also converted to the Aronchick scale. In several publications these scales had not been strictly defined and in a conservative approach only a conversion to broad categories (optimal/ suboptimal or adequate/ inadequate) was done.

**Table 1:** Bowel preparation scales and definitions used.

**Aronchick scale** [17]
excellent: a small volume of clear liquid or >95% of surface seen
good: large volume of clear liquid covering 5–25% of the surface but >90% of surface seen
fair: some semisolid stool, >90% of surface seen
poor: semisolid stool could not be sucked away, <90% of surface seen
inadequate: repeat preparation needed
**Boston Bowel Preparation Scale (BBPS)** [33, 40]
Score 3 segments of the colon
0: Unprepared colon segment with mucosa not well seen due to solid stool that cannot be cleared
1: Portion of mucosa of the colon segment seen, but other areas are not well seen due to staining, residual stool and/ or opaque liquid
2: Minor amount of residual staining, small fragments of stool and/ or opaque liquid but mucosa of colon segment well seen
3: Entire mucosa of the colon well seen with no residual staining, small fragments of stool and/ or opaque liquid. *Comment*:
*score reaches from 0–9 (for each colon segment separate scores*, *e*.*g*., *2+2+3 = 7)*
**Other definitions**
Optimal = good + excellent
Suboptimal = insufficient + poor + fair
Adequate = fair + good + excellent
Inadequate = insufficient + poor

### Quality of included studies

The quality of all included studies was evaluated following a strategy adapted from a previous meta-analysis [16]: *i) Adenoma detection*: 1 point was given if adenoma detection and 0 points if only polyp detection had been reported. *ii) Study population*: 2 points if the study population consisted only of patients referred for CRC screening, 1 point if only patients for CRC screening and adenoma surveillance were considered, and 0 points if patients with symptoms or indications other than CRC screening and adenoma surveillance were also included. *iii) Study design*: 1 point for a prospective, 0 point for a retrospective study design. *iv) Bowel preparation*: 1 point if training, exercises or internal validations for the respective bowel preparation scale had been performed. *v) Confounders*: 2 points were given if the study controlled for all crucial confounders: age, gender and colon withdrawal time. 1 point if the study controlled for any confounders.

### Statistical analysis

#### Meta-analysis

**Fig 2:**
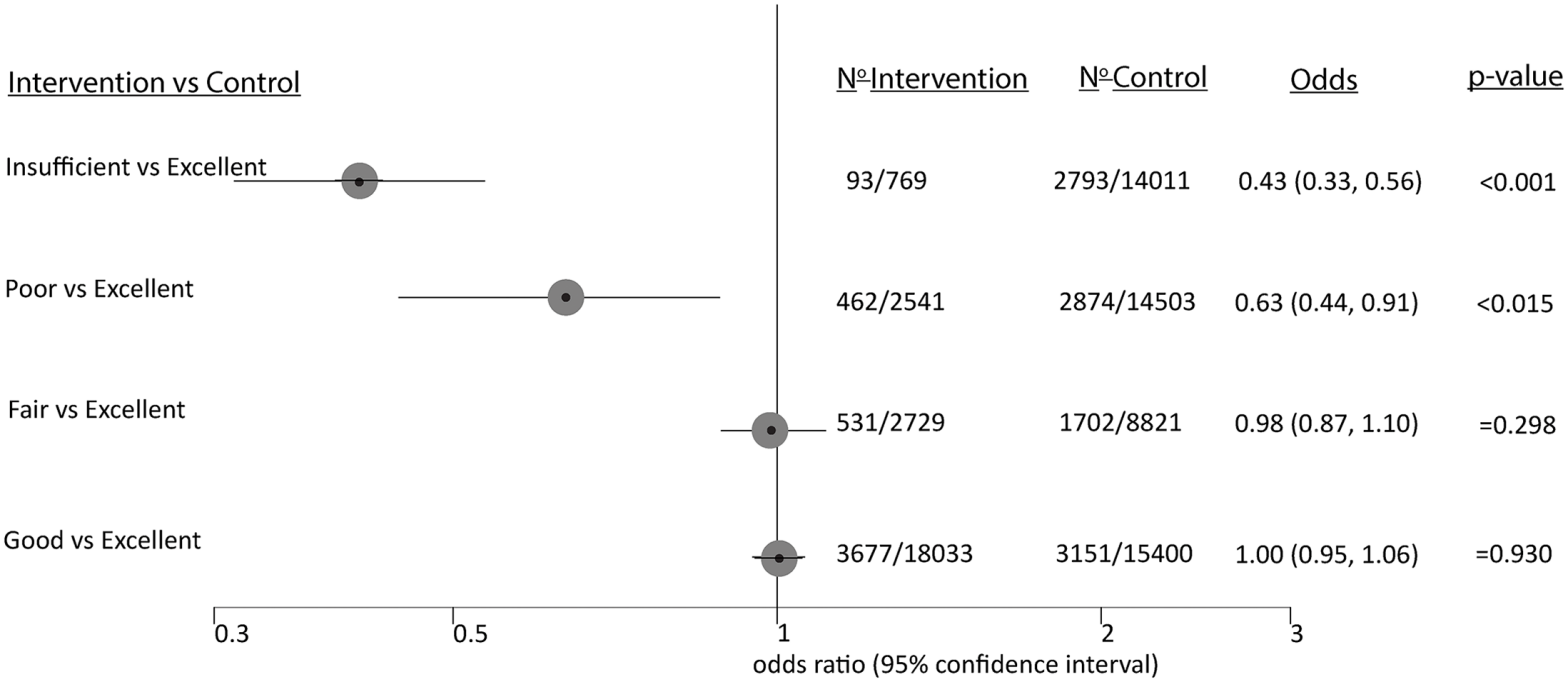
Effects of insufficient, poor, fair and good bowel preparation compared to an excellent preparation on overall detection of colonic lesions in a network meta-analysis.

The meta-analysis was performed to summarize early and advanced adenoma detection in all studies included. Unless otherwise mentioned, meta-analysis was performed comparing two kinds of bowel preparations at a time. For this pairwise comparison, the total number of subjects who had either of the two described qualities of preparation as well as the number of patients for whom at least one adenoma was detected was tabulated. The number of studies included as well as the number of patients in these sub-categories are reported (Fig 2 and S1 Fig). For our meta-analysis we used a random-effects model weighing the contributions from different studies based on both the intra-study and inter-study variances. StatsDirect software version 3.0.150 (StatsDirect Ltd., Sale, Cheshire, UK; www.statsdirect.com) was used and Forest plots as well as funnel plots were generated. The overall OR, confidence intervals (CI), and p-values obtained from a random-effects model were reported. Bias assessment graphs were also generated but indicated a lack of bias according to visual inspection (S2 Fig). The number of studies included was not sufficient to obtain a reliable bias-estimator such as the Begg-Mazumdar estimator (typically requiring more than 25 high quality studies) and no such analysis was performed.

We performed a network meta-analysis to summarize adenoma detection according to the five qualities of the Aronchick scale with pairwise comparison of all 5 preparation qualities. For these calculations we used the algorithms by Chaimani et al. [20] and the corresponding STATA modules (http://www.mtm.uoi.gr). The analysis showed indirect effects among the categories to be irrelevant. We therefore reported the pairwise meta-analysis results relative to good or excellent preparation.

## Results

Our systematic literature research identified 204 potentially relevant studies which were screened for eligibility; 27 of those fulfilled our inclusion criteria and were used in subsequent analyses (Table 2). We included studies of two different categories: i) 21 comparative (observational) studies for which adenoma/polyp detection rates were compared according to bowel preparation within given study populations, ii) 6 repeat-colonoscopy studies in which results of a second colonoscopy (tandem colonoscopy) after an initial endoscopy with less than optimal preparation were reported. Due to different study designs both types of studies are summarized separately.

**Table 2:** Summary of all included studies.

Study Publication year	Quality points	Number (n) colonoscopies	Study type	Bowel preparation scale
Mahadev [1] 2014	4	1,649	Comparative Retrospective	Unique
Kim [28] 2014	5	482	Comparative Prospective	BBPS Aronchick
Holt [19] 2014	4	413	Comparative Prospective	Ottawa
Singhal [23] 2014	1	297	Repeat-colonoscopy Retrospective	Aronchick
Anderson [25] 2014	1	13,022	Comparative Prospective	Unique
Lee [41] 2014	4	31,088	Comparative Prospective	Unique
Fayad [42] 2013	2	2,163	Comparative Retrospective	Aronchick
Menees [31] 2013	3	71	Repeat-colonoscopy Retrospective	Aronchick
Gao [37] 2013	4	1,012	Comparative Prospective	BBPS
Jover [26] 2013	6	4,539	Comparative Prospective (RCT)	Aronchick
Bryant [27] 2012	0	1,785	Comparative Retrospective	Unique
Adler [43] 2013	6	11,166	Comparative Prospective	Aronchick-based
Chokshi [29] 2012	3	133	Repeat-colonoscopy Retrospective	Aronchick
Goncalves [44] 2011	2	1,545	Comparative Retrospective	Unique
Sherer [45] 2012	2	8,800	Comparative Retrospective	Unique
De Jonge [39] 2012	2	4,800	Comparative Retrospective	Unique
Enestvedt [46] 2011	4	190	Comparative Prospective (RCT)	BBPS
Calderwood [33] 2010	4	983	Comparative Prospective	BBPS
Shaukat [47] 2009	5	47,253	Comparative Retrospective	Unique
Radaelli [48] 2008	1	12,835	Comparative Consecutive	Unique
Froehlich [22] 2005	2	5,832	Comparative Prospective	Unique
Harewood [13] 2003	1	93,004	Comparative Retrospective	Unique
Pontone [49] 2014	1	190	Comparative Retrospective	Aronchick
Xiang [30] 2014	3	2,093	Repeat-colonoscopy Retrospective	Unique
Aslanian [50] 2013	4	502	Comparative Prospective (RCT)	Aronchick
Lebwohl [12] 2011	1	216	Repeat-colonoscopy Retrospective	Aronchick
Hong [21] 2012	5	277	Repeat-colonoscopy Prospective	Aronchick

Quality points were given for a detailed reporting of lesions, the study population, study design, validation of stool scales and correction for confounders (for details see methods). RCT: Randomized controlled trial. BBPS: Boston Bowel Preparation Scale.

### Bowel preparation and overall adenoma and polyp detection

We included 21 comparative studies in our meta-analysis. Aronchick scale was used in 5 of these studies to report bowel preparation quality, 1 study used an Aronchick-based scale, 3 studies used BBPS, and 1 the Ottawa scale. 11 additional studies used unique non-validated scales. Bowel preparation quality was converted to the 5 qualities of the Aronchick scale (“excellent”, “good”, “fair”, “poor”, “insufficient”), or two pairs of broader categories “optimal” (i.e. good/excellent) vs. “suboptimal” (i.e. fair/poor/insufficient) and “adequate” (i.e. excellent/good/fair) vs. “inadequate” (i.e. poor/insufficient). When bowel preparation quality of all studies was summarized, 77% of patients had optimal preparation, and 95% had adequate preparation.

**Fig 3:**
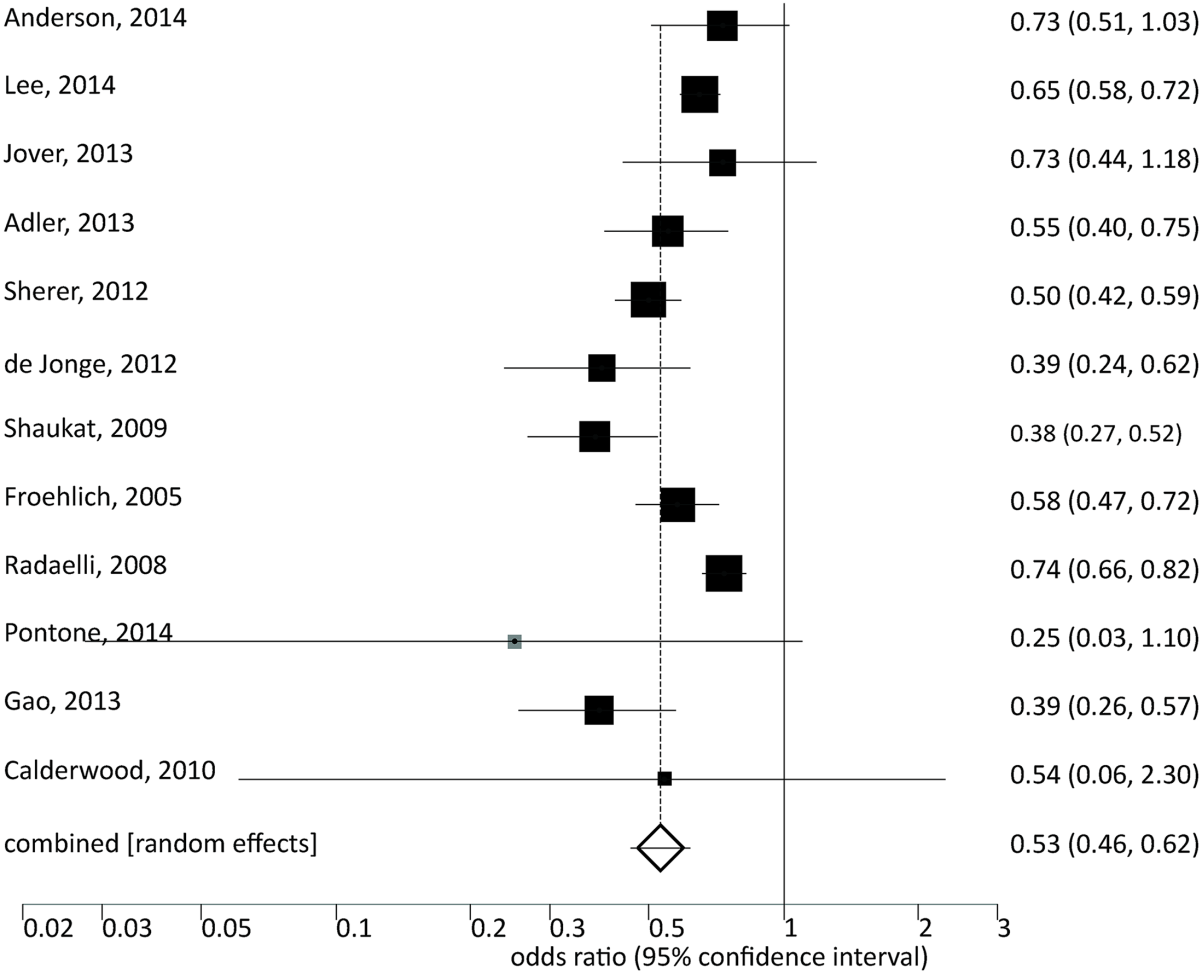
Meta-analysis of studies showing effects of *inadequate vs*. *adequate* bowel preparation regarding detection of colonic lesions.

Low-quality bowel preparation was significantly associated with reduced detection of polyps or adenomas (Fig 3 and S3 Fig): For inadequate vs. adequate preparation the OR was 0.53 (CI: 0.46–0.62, p<0.001). For suboptimal vs. optimal preparation an OR of 0.81 (CI: 0.74–0.89, p<0.001) was calculated.

We also summarized adenoma detection for the *individual* qualities of the Aronchick scale (Fig 2). Despite no difference for excellent, good, and fair bowel preparation adenoma detection decreased with poor and insufficient preparation (poor: OR 0.63, CI: .44–0.91, p<0.015; insufficient: OR 0.43, 0.33–0.56, p<0.001). However, only four studies provided data regarding insufficient bowel preparation.

For a sensitivity analysis we assessed the quality of the included studies (see legend of Table 2 and methods section). When we restricted our analysis to high-quality studies (8 studies with ≥4 quality points) our conclusions remained intact (OR suboptimal vs. optimal: 0.76, CI 0.69–0.83, p<0.001; OR inadequate vs. adequate: 0.57, CI 0.46–0.72, p<0.001; S1 Table). Similarly, restriction to studies with adenoma (not polyp) detection or to studies using Aronchick scale/ Aronchick based scale or a defined scale did not change our conclusions.

No publication bias was detected and all funnel plots for all analyses looked symmetric (see S1 Fig and data not shown).

### Detection of advanced adenomas/ large polyps

**Fig 4:**
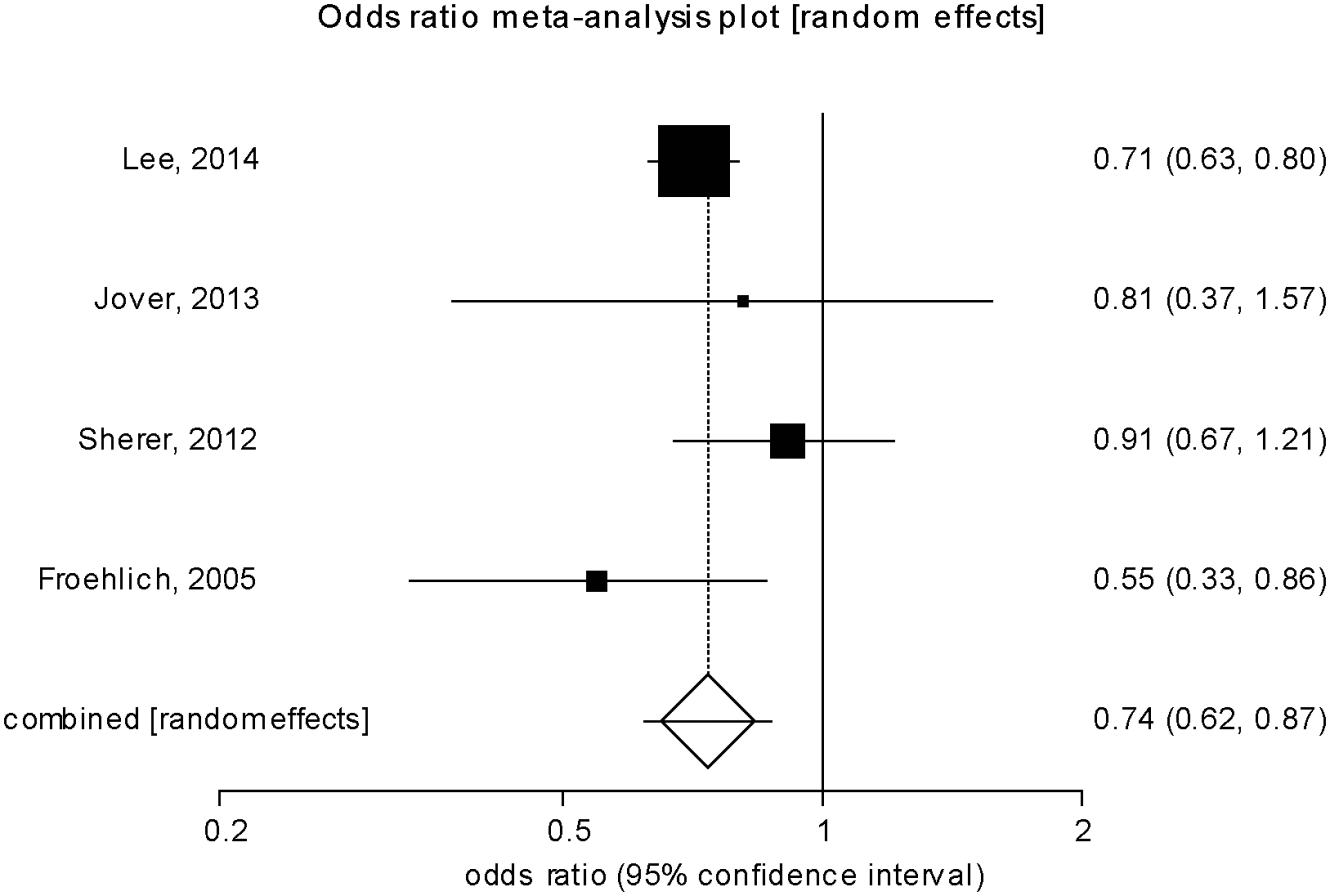
Meta-analysis of *inadequate and adequate* bowel preparation regarding detection of *advanced adenomas or polyps*.

In comparative studies, low-quality bowel preparation was also associated with reduced detection of *advanced* lesions. In this analysis 7/11 studies defined advanced polyps by histological information, 4/11 studies by polyp size >9mm. Inadequate vs. adequate preparation significantly reduced detection of advanced lesions (OR 0.74, CI: 0.62–0.87, p<0.001; Fig 4). In comparison to early advanced colonic lesions (see above), advanced lesions are affected stronger by inadequate preparation showing non-overlapping confidence of the OR for early and advanced lesions.

Suboptimal vs. optimal preparation showed a strong trend for reduced lesion detection (OR 0.94, CI: 0.87–1.01, p = 0.33; data not shown). When we compared the 5 preparation qualities of the Aronchick scale, detection of advanced lesions tended to be lower for poor and insufficient bowel preparation (poor vs. good/excellent: OR 0.79; CI: 0.53–1.19, p = 0.259; insufficient vs. good/excellent: OR 0.75; CI: 0.27–2.14, p = 0.777; S1 Fig). However, the low number of lesions (85 for poor, 4 for insufficient) limits our conclusions.

In a sensitivity analysis our conclusions remained robust when studies with and without histological information were distinguished (inadequate vs. adequate: OR advanced adenoma detection 0.76, CI: 0.65–0.89, p<0.001; OR advanced polyp detection: 0.55, CI: 0.33–0.86, p = 0.006; S2 Table). Similarly, our conclusions did not change when only high-quality studies or studies using a defined scale were considered.

### Detection of early and advanced adenomas in repeat-colonoscopy studies

We included 5 repeat-colonoscopy studies (Table 3). The heterogeneous study design precluded direct comparison and meta-analysis. Results of some were reported as adenoma miss rates, defined as the number of adenomas detected during the second colonoscopy divided by the sum of all adenomas in the first and second colonoscopy. Adenoma miss rates ranging from 27%-56% for colonoscopies with poor, fair or suboptimal preparation were reported. Only one study provided an internal control with colonoscopies of good and excellent preparation with adenoma miss rates of 27% and 21%, respectively [21].

**Table 3:** Summary of repeat-colonoscopy studies.

Study Publication year	Quality points	Study population Number of included patients	Number lesions analyzed	Preparation first colonoscopy	Preparation Second Colonoscopy	Time Repeat colonoscopy	Adenoma miss rate (ADR)	Advanced adenoma miss rate (Adv. ADR)
**Studies providing analysis *per adenoma* (adenoma miss rate)**								
Menees [3] 2013	3	619 Screening colonoscopies. 71 included	A: 163 AdvA: 22	Fair	optimal: 58%,	<3y	31%	0%
					fair: 21%,			
					poor: 20%			
Chokshi [29] 2012	3	373 Screening colonoscopies. 133 included	A: 190 AdvA: 30	Poor: 87%	Adequate: 77%,	Mean 340 days	48%	50%
				Insufficient: 13%	inadequate: 23%			
Lebwohl [12] 2011	2	3047 Screening and diagnostic colonoscopies. 216 included	A: 198	Suboptimal	Excellent/Good	<3 y	Poor: 56%	Poor: 29%
							Fair: 42%	Fair: 26%
							Screening: 43%	Screening: 37%
Hong [21] 2012	5	Patients scheduled for polypectomy after a first diagnostic colonoscopy.277 included	A: 714 AdvA: 184	Excellent: 32%	Excellent/ good	<3 mo	Excellent: 21%	Excellent: 9%
				Good: 41%			Good: 27%	Good: 17%
				Fair: 20%			Fair: 27%	Fair: 18%
				Inadequate: 7%			Inadequate: 47%	Inadequate: 37%
**Studies providing analysis *per patient* (ADR, Adv. ADR)**								
Singhal [23] 2014	1	10908 colonoscopies of screening and diagnostic indications 297 included		Inadequate	Optimal: 52%	<5y	First colonoscopy22%	First colonoscopy: 7.4%
					fair: 31%,		Repeat- colonoscopy 4%	Repeat-colonoscopy 8.4%
					poor: 17%			

A: adenoma, AdvA: advanced adenoma, ADR: adenoma detection rate, AdvA. ADR: advanced adenoma detection rate

*Advanced* adenoma miss rates ranged from 0–50%. Only one study reported an internal control regarding advanced adenoma detection in a repeat-colonoscopy after a colonoscopy of excellent or good preparation reporting a miss rate of 9% and 17%, respectively [21].

All repeat-colonoscopy studies reported on a selected patient population: repeat examinations were either performed in a small subset of the original study population (Table 3) or only patients scheduled for polypectomy were included [21].

### Carcinoma detection

Of the comparative studies only one large study with 5832 patients [22] reported on differential carcinoma detection. In this study a non-significant paradoxical trend of lower carcinoma detection in patients with optimal vs. inadequate preparation was noted (OR 0.68; CI: 0.45–1.02, p = 0.063). All of the remaining comparative studies were either underpowered (reporting no or only 1 carcinoma case) or did not comment on carcinoma detection.

In one repeat-colonoscopy study with 150 repeat examinations, three carcinoma cases had been missed during the initial investigations and were detected during the second colonoscopy [23]. However, all carcinomas were detected in symptomatic patients and no carcinoma had been missed in patients referred for CRC screening.

### Detection of right-sided, flat or serrated adenomas

We identified 11 studies addressing differential detection of right-sided, flat or serrated adenomas in patients with low quality bowel preparation (Table 4). 7 studies were comparative, 4 studies were repeat-colonoscopy studies. A heterogeneous study design and different end-points precluded a meta-analysis.

**Table 4:** Effects of bowel preparation on the detection of right-sided, flat or serrated polyps/ adenomas.

Study Year of publication	Quality	n	Readout	Study results	Study conclusion
***Comparative* studies**					
Kim [28] 2014	5	482	Right- sided polyps	Correlation analysis: BBPS≥ 8 vs. BBPS< 8 BBPS segment scores in the right colon but not in the left colon correlated with polyp detection rates (right: r = 0.107, p = 0.018; left: r = 0.059, p = 0.198)	Bowel preparation **more** relevant for polyp detection in the **right** colon
de Wijkerslooth [24] 2013	6	1,354	Proximal serrated adenoma	Multivariate analysis: Ottawa scale was associated with:	Bowel preparation **equally** relevant for detection of right-sided serrated adenoma vs. other adenomas
				- overall ADR (OR 0.95; CI: 0.91–0.99)	
				- but not proximal SDR (OR: 0.98; CI: 0.92–1.05)	
Anderson [25] 2014	1	13,022	Right- sided serrated	Optimal vs. poor bowel preparation	Bowel preparation **equally** relevant for overall, right-sided, and serrated adenoma detection
				- Overall ADR: 26.3% vs. 20.9%	
				- Proximal ADR: 12.9% vs. 8%	
				- SDR 8.8% vs. 7.5%	
				no significant differences	
Lee [41] 2014	4	31,088	Right-sided	Adequate vs. inadequate bowel preparation	Bowel preparation **equally** relevant for adenoma detection in the right and left colon
				- OR overall adenoma detection 1.38 (1.23–1.54)	
				- OR right sided adenoma 1.16 (1.13–1.33)	
				no significant differences	
Jover [26] 2013	6	4,539	Right-sided	No significant effects of bowel preparation on adenoma detection, similar trends for right- and left sided adenomas	Bowel preparation **equally** relevant for adenoma detection in the right and left colon
Bryant [27] 2012	0	1785	Right- sided polyp	Adequate vs. poor preparation	Bowel preparation **equally** relevant for polyp detection in the right and left colon
				- OR left-side: 1.1 (0.8–1.5)	
				- OR right-side: 1.1 (0.8–1.5)	
				no significant differences	
Calderwood [33] 2010	4	983	Right-sided polyp	BBPS 0.1 vs. BBPS 2,3. Multivariate analysis	Bowel preparation **equally** relevant for polyp detection in the right and left colon
				- OR right side: 1.6 (1.01–2.55)	
				- OR left side: 2.6 (1.34–4.98)	
***Repeat* colonoscopy studies**					
Xiang [30] 2014	3	2,093	Flat adenoma	In patients with poor bowel preparation the OR for missing a flat adenoma is 4.4 (no comparison to protruding adenoma provided)	Bowel preparation relevant for **flat** adenoma miss rate
Lebwohl [12] 2011	1	216	Proximal	Adenoma miss rate 42% for proximal and distal adenomas	Bowel preparation **equally** relevant for adenoma miss rate in the right and left colon
Singhal [23] 2014	1	297	Right-sided	67% of all missed adenomas were right-sided adenomas	Bowel preparation **more** relevant for adenoma miss rate in the **right** colon
Chokshi [29] 2012	3	133	Right-sided	65% of all missed adenomas and 80% of all missed advanced adenomas were in the right colon	Bowel preparation **more** relevant for adenoma miss rate in the **right** colon

n.s. = not significant. ADR = Adenoma detection rate. Adv. ADR = Advanced adenoma detection rate. SDR = serrated adenoma detection rate. BBPS = Boston Bowel Preparation Scale.

As shown in Table 4, 6 out of 7 comparative studies [24–27] failed to detect a disproportionate effect of low bowel preparation for the detection of proximal and/ or serrated lesions. In only one study low quality preparation was associated with reduced right-sided but not left-sided polyp detection [28]. However, this study was the smallest of all comparative studies addressing this question.

In contrast, in repeat-colonoscopy studies less than optimal preparation was associated with diminished detection of flat/ serrated or right-sided lesions. In two studies approximately 65% of all missed lesions were right-sided and even 80% of all advanced adenomas missed resided in the right colon [23,29]. One tandem colonoscopy study noted an OR of 4.4 for flat adenomas compared to protruding adenomas to be missed in a poorly prepared colon [30]. In one repeat-colonoscopy study bowel preparation effected adenoma detection in the left and right colon in a similar manner [12].

## Discussion

We performed a systematic literature research and meta-analysis to distinguish effects of low-quality bowel preparation on the differential detection of early, advanced colonic lesions and cancer during colonoscopy. We found that with inadequate bowel preparation, the chance of detecting early vs. advanced polyps drops by 44% and 23%, respectively. With suboptimal preparation, detection of early lesions is reduced by 20%, advanced lesions also tend do be detected less frequently. Due to consideration of a larger number of original studies and subjects, our meta-analysis demonstrates stronger effects of inadequate bowel preparation on early vs. advanced lesions which have not been apparent in a previous meta-analysis [16].

Our analysis was based on 21 studies summarizing 247,277 colonoscopies regarding overall detection of colonic lesions and 10 studies summarizing 122,958 colonoscopies regarding advanced lesions. Our and a previous meta-analysis [16] thus provide reliable estimates regarding the likelihood of missing adenoma and advanced adenomas.

In contrast, few studies addressed *carcinoma* detection in patients with low-quality bowel preparation. Endoscopists might feel assured that at least a cancer would have been detected but no direct evidence exist to back up such a claim. Carcinoma detection will likely be less effected than detection of advanced adenomas; however, the frequency of missed carcinoma cases remains unknown. Our study also identifies a lack of data regarding adenoma detection in the insufficiently prepared colon.

The risk of missing early and advanced adenomas has also been addressed by repeat-colonoscopy studies [12,21,23,29,31] (Table 3), which was consistently higher than for comparative studies. There are several explanations for this discrepancy: i) 8–35% of early and 5–10% of advanced adenomas are missed even in a perfectly prepared colon [21,32] and this baseline adenoma miss rate would need to be subtracted from the numbers in Table 3. ii) Only a fraction of all patients eligible for repeat colonoscopy effectively underwent a repeat endoscopy. This might constitute a strong selection bias for high-risk patients. iii) Some studies reported a significant time interval between the first and second colonoscopy, allowing new lesions to appear. Comparing to repeat-colonoscopy studies, results of comparative studies were more homogenous, supported by a larger number of patients and individual studies, and seem to have higher credibility at least for patients at average risk.

After screening colonoscopy with low-quality preparation many gastroenterologists recommend an early repeat-colonoscopy [33]. However, since a significant fraction of all colon lesions will have been detected and removed during the initial exam the cost-efficiency of an immediate second endoscopy will be strongly reduced. It should be noted that even with inadequate preparation considerable efficacy of colonoscopy remains: Inadequate preparation reduces detection of overall lesions by 47% and advanced lesions by only 26%. In our opinion, our results thus argue for a repeat-colonoscopy after a delay of several years. Ultimately a cost-effectiveness analysis considering effects on both, early and advanced lesions will be needed to determine the optimal time interval of a re-colonoscopy. Clearly, different considerations apply to a symptomatic patient with a high pre-test probability of advanced lesions as these patients will likely benefit from an immediate second exam. In one repeat-colonoscopy study all patients with carcinoma that were missed in the initial colonoscopy were symptomatic patients [23].

It has been suggested that colon preparation disproportionally affects detection of flat, serrated and/or right-sided adenomas as these preferentially reside in the right colon [34] and are more difficult to detect by colonoscopy [14,15]. Therefore, these lesions might be responsible for a significant fraction of right-sided interval carcinomas. However, in our analysis the majority of all comparative studies did not find significantly diverging detection rates for adenoma subtypes in patients with different qualities of bowel preparation. In contrast, a number of small repeat colonoscopy studies described lower detection rates of right-sided lesions in a poorly prepared colon. This discrepancy might be explained by the strong selection bias in repeat colonoscopy studies with only a small fraction of all included patients undergoing repeat examinations. In addition, a long time interval (>1 year) between the index and repeat exam would favor fast growing lesions and growth rates of right-sided/flat/serrated might differ compared to pedunculated adenomas. However, one large tandem colonoscopy study described a more than 4-fold higher chance of missing a flat compared to a penduculated adenoma suggesting that detection rate differs if the analysis focuses on endoscopic appearance rather than histology or location within the colon [30]. More data are clearly needed to answer this important question.

The predictive value of preparation quality for reduction of adenoma detection will depend on inter- and intraobserver agreement as well as validity of the scale used for the description of bowel preparation. The Aronchick scale was the first scale for standardized assessment of bowel preparation [17,35]. BBPS uses similar wording as the Aronchick scale but combines 4 qualities of 3 colon segments to a single score (0–9). For BBPS online training material is available (http://www.cori.org/bbps/). The Ottawa and Chicago scale also provide semi-quantitative measurements for bowel preparation [36]. However, these scales cannot be directly converted to the Aronchick or BBPS scale since preparation quality is evaluated after sucking away all liquid material and fluid in the colon is also penalized.

Internal validation of preparation quality has not been done in most of the included studies. However, after several training sessions near-perfect interrater agreement can be achieved for BBPS [18,33,37] as well as Aronchick scale and Chicago scale [36]. In clinical practice, quality of bowel preparation should be reported for each colonoscopy as suggested by several guidelines but reporting of bowel preparation in daily practice is sometimes incomplete or suboptimal [38,39].

Our analysis has some limitations: i) The literature research was restricted to publications in English. ii) Bowel preparation has not been uniformly evaluated by a standardized scale with frequent usage of unique preparation scales. In these cases transformation to the Aronchick scale leaves some ambiguity. Moreover, most studies did not perform internal validation of the respective preparation scale. iii), while robust data could be retrieved regarding overall detection of lesions for fair and poor bowel preparation, few data exist regarding insufficient bowel preparation and detection of advanced lesions at the lower end of the preparation scale. Finally, most of the studies did not report or remained underpowered to detect enough CRC to make reliable predictions regarding missed carcinomas after low-quality preparation.

In conclusion our analysis demonstrates a stronger drop in early vs. advanced adenoma detection in the inadequately prepared colon. Therefore, considerable efficacy of colonoscopy even with inadequate bowel preparation remains since the majority of advanced lesions will still be detected. Therefore, cost-effectiveness studies will be needed to determine the best strategy for repeat-endoscopy in this situation.

## Supporting Information

S1 Fig Effects of insufficient, poor and fair bowel preparation compared to a good or excellent preparation on the detection of *advanced* lesions in a network meta-analysis. (TIFF)

S2 Fig Funnel plot for the studies showing a difference between *the sub-optimal and optimal* bowel preparation regarding overall detection of colonic lesions. (TIFF)

S3 Fig Meta-analysis regarding detection of early lesions (adenomas or polyps) with *sub-optimal vs*. *optimal* bowel preparation. (TIF)

S1 File Literature search strategy. (PDF)

S1 Table Sensitivity analysis for the meta-analysis regarding overall detection of adenoma/ polyps. This analysis was performed using several sub-studies with different inclusion criteria. The table shows the analyses on the studies reporting the ORs for *suboptimal vs*. *optimal* bowel preparation, considering any adenoma + any polyp.
(DOC)

S2 Table Sensitivity analysis for the meta-analysis regarding detection of advanced adenomas/ polyps. This table shows the analyses on the studies reporting the odds for *inadequate vs*. *adequate* bowel preparation, considering *advanced adenomas + advanced polyps*.
(DOC)

## Abbreviations

ADR: Adenoma detection rate
Adv ADR: Advanced adenoma detection rate
BBPS: Boston bowel preparation scale
CRC: Colorectal cancer
CI: Confidence intervals
N.s.: not significant
OR: Odds Ratio
SDR: serrated adenoma detection rate
