# Supplementary figures and images for: Meta-Analysis of the Effect of Bowel Preparation on Adenoma Detection: Early Adenomas Affected Stronger than Advanced Adenomas
Source: PLoS One. 2016 Jun 3;11(6):e0154149. doi: 10.1371/journal.pone.0154149 (PMC4892520; doi:10.1371/journal.pone.0154149)

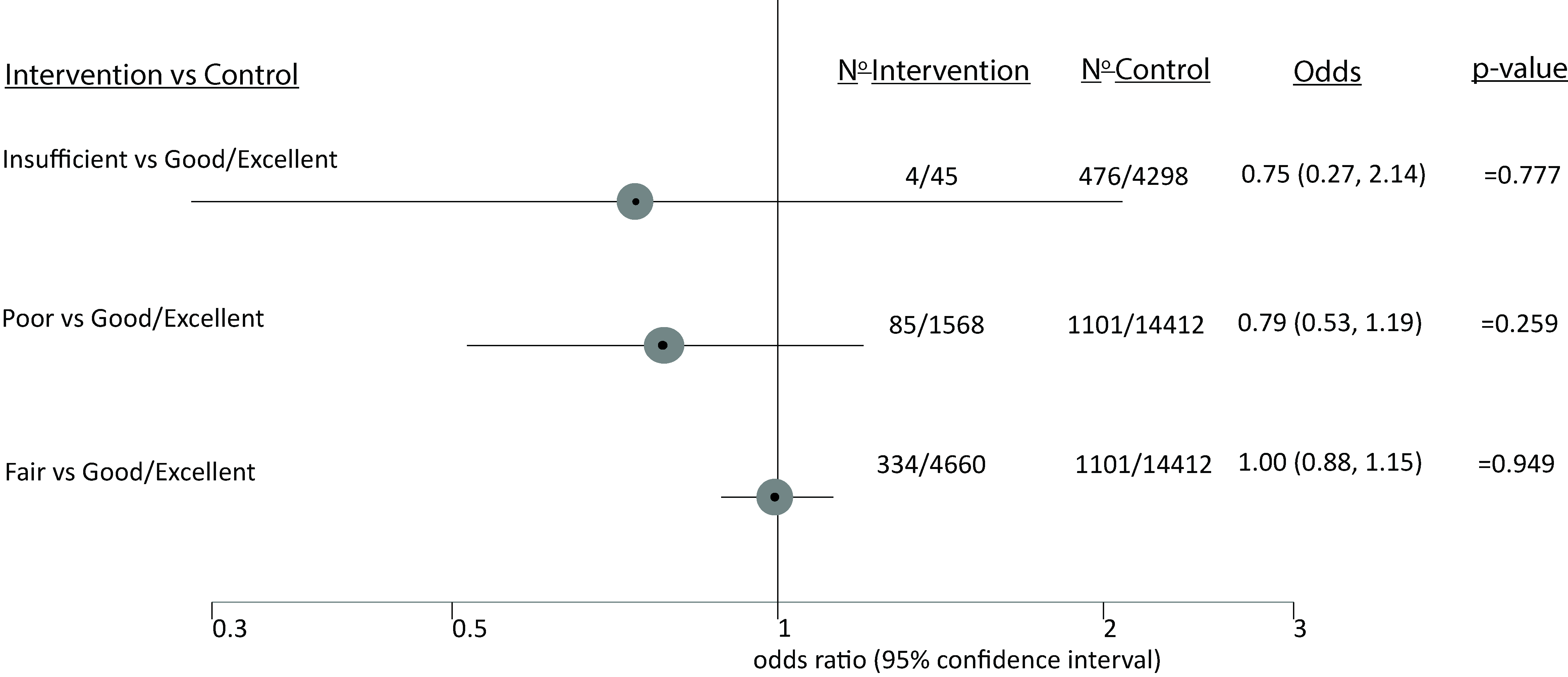

Supplement: S1 Fig — (TIFF) [file pone.0154149.s001.tiff]

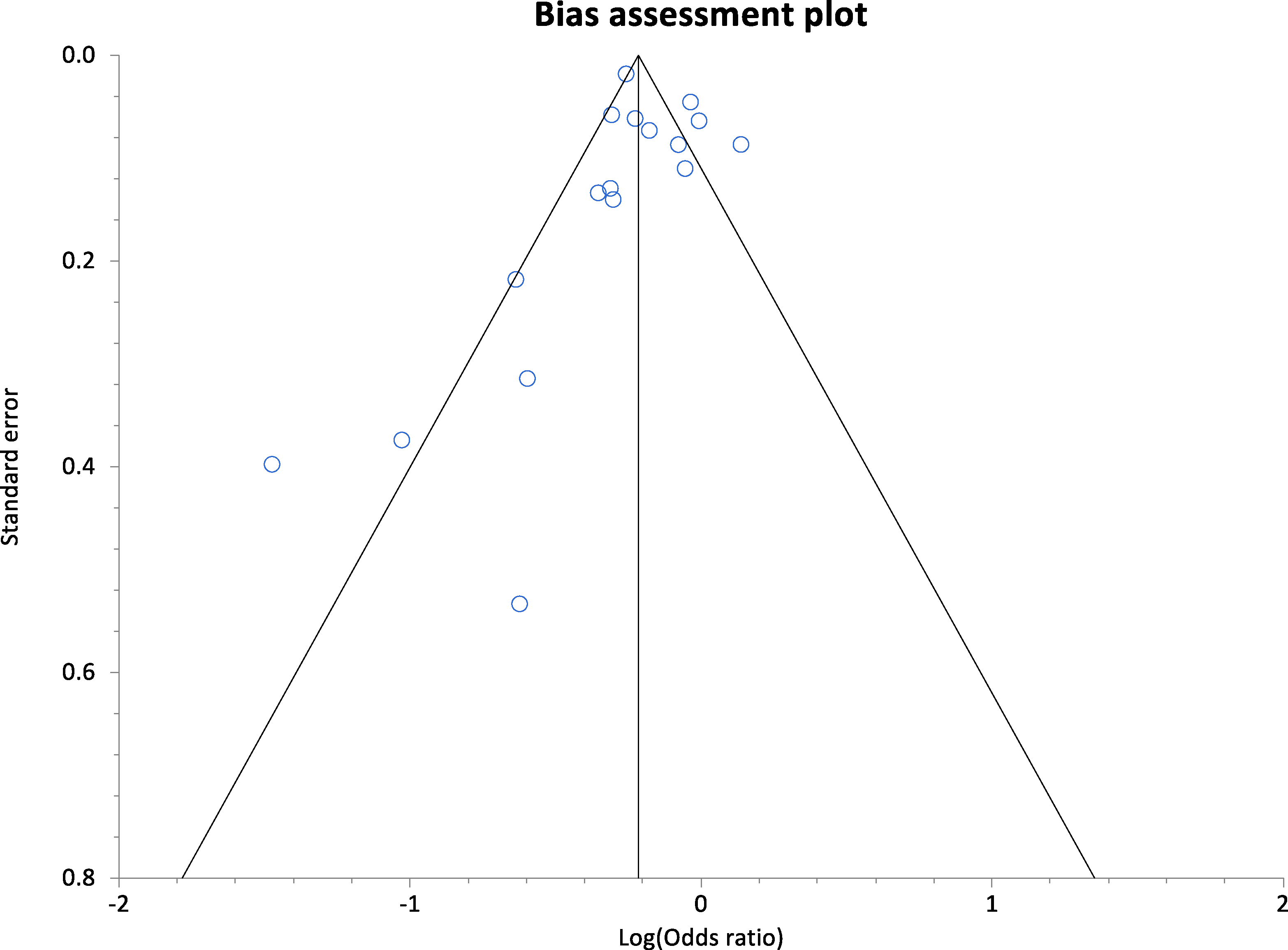

Supplement: S2 Fig — (TIFF) [file pone.0154149.s002.tiff]
