# Supplementary material for: Meta-Analysis of the Effect of Bowel Preparation on Adenoma Detection: Early Adenomas Affected Stronger than Advanced Adenomas
Source: PLoS One. 2016 Jun 3;11(6):e0154149. doi: 10.1371/journal.pone.0154149 (PMC4892520; doi:10.1371/journal.pone.0154149)
Supplement: S1 File — (PDF) [file pone.0154149.s004.pdf]

Literature search strategy

We identified relevant studies via an electronic search using MEDLINE. Our MEDLINE search was performed until November 7th, 2014 (date last searched). Our electronic searches were supplemented by manual scanning of the reference lists of all relevant studies and review articles. MEDLINE search combined search terms related to bowel preparation, adenoma and polyp detection, colonoscopy and bowel preparation scales.

The following individual searches were performed:

- (Adenoma detection OR polyp detection) AND bowel preparation (95)
- colonoscopy AND Aronchick scale (29)
- colonoscopy AND Ottawa scale (44)
- colonoscopy AND (Boston bowel preparation scale OR BBPS) (25)
- tandem colonoscopy AND (adenoma OR polyp) (47)
