# Supplementary material for: Meta-Analysis of the Effect of Bowel Preparation on Adenoma Detection: Early Adenomas Affected Stronger than Advanced Adenomas
Source: PLoS One. 2016 Jun 3;11(6):e0154149. doi: 10.1371/journal.pone.0154149 (PMC4892520; doi:10.1371/journal.pone.0154149)
Supplement: S1 Table — This analysis was performed using several sub-studies with different inclusion criteria. The table shows the analyses on the studies reporting the ORs for suboptimal vs. optimal bowel preparation, considering any adenoma + any polyp. (DOC) [file pone.0154149.s005.doc]

| ***All* adenomas: *Sub-optimal vs. optimal* bowel preparation** | | | | | |
| --- | --- | --- | --- | --- | --- |
| **Sub-criterion for inclusion** |  | **Combined OR** | **Number of studies** | **Patients sub-optimal** | **Patients optimal** |
| **All** | All | 0.82; CI: 0.74-0.89, p<0.001 | 17 | 36,666 | 126,837 |
| **Quality** | High quality | 0.76; CI: 0.69-0.83 p<0.001 | 8 | 10,046 | 34,205 |
|  | Low quality | 0.86; CI: 0.76-0.97 p=0.018 | 9 | 26,620 | 92,632 |
| **Adenoma or polyp** | Only adenomas | 0.82; CI: 0.73-0.92, p<0.001 | 14 | 13,368 | 50,315 |
|  | Only polyps | 0.77; CI: 0.70-0.83, p<0.001 | 3 | 23,298 | 76,522 |
| **Validation** | Validated | 0.50; CI: 0.29-0.85, p=0.011 | 3 | 35,923 | 125,198 |
|  | Not validated | 0.84; CI: 0.77-0.92, p<0.001 | 14 | 743 | 1639 |
| **Type of scale** | BBPS or Aronchick based | 0.70; CI: 0.56-0.87, p=0.002 | 9 | 4,512 | 19,377 |
|  | Any other scale | 0.83; CI: 0.76-0.91, p<0.001 | 8 | 32,154 | 107,460 |
