# Supplementary material for: Meta-Analysis of the Effect of Bowel Preparation on Adenoma Detection: Early Adenomas Affected Stronger than Advanced Adenomas
Source: PLoS One. 2016 Jun 3;11(6):e0154149. doi: 10.1371/journal.pone.0154149 (PMC4892520; doi:10.1371/journal.pone.0154149)
Supplement: S2 Table — This table shows the analyses on the studies reporting the odds for inadequate vs. adequate bowel preparation, considering advanced adenomas + advanced polyps. (DOC) [file pone.0154149.s006.doc]

| ***Advanced* adenomas + polyps: *Inadequate vs. adequate*** | | | | | |
| --- | --- | --- | --- | --- | --- |
|  |  | **Combined OR** | **Number of studies** | **Patients inadequate** | **Patients adequate** |
| **All** | All | 0.74; CI: 0.62-0.87 p<0.001 | 4 | 3,173 | 46,891 |
| **Quality** | High quality | 0.71; CI: 0.63-0.80 p<0.001 | 2 | 1,743 | 33,689 |
|  | Low quality | 0.73; CI: 0.44-1.19 p=0.037 | 2 | 1,430 | 13,202 |
| **Adenoma or polyp** | Only adenomas | 0.76; CI: 0.65-0.89 p<0.001 | 3 |  |  |
|  | Only polyps | 0.55; CI: 0.33-0.86 p=0.006 | 1 | 601 | 5,231 |
| **Validation** | Validated | - | 0 |  |  |
|  | Not validated | 0.74; CI: 0.62-0.87 p<0.001 | 4 (same as overall) | 3,173 | 46,891 |
| **Type of scale** | BBPS or Aronchick based | 0.81; CI: 0.37-1.57 p=0.640 | 1 | 95 | 4,435 |
|  | Any other scale | 0.73 ; CI: 0.59-0.91 p<0.001 | 3 | 3,078 | 42,456 |
